# Sexual behavior and knowledge of human immunodeficiency virus/aids and sexually transmitted infections among women inmates of Briman Prison, Jeddah, Saudi Arabia

**DOI:** 10.1186/1471-2334-14-290

**Published:** 2014-05-24

**Authors:** Wafa MK Fageeh

**Affiliations:** 1 Department of Obstetrics and Gynecology, King Abdulaziz University, King Abdullah Road, PO Box 80215, Jeddah 21589, Saudi Arabia

## Abstract

**Background:**

To reduce the incidence of HIV and sexually transmitted infections (STIs), it is necessary to target high-risk populations such as prison inmates. This study aims to explore the range of knowledge on HIV and STIs, sexual behaviors, and adoption of preventive measures among women inmates.

**Methods:**

This was a survey conducted between July 1, 2012 and July 29, 2012 among women inmates at Briman Prison, Jeddah, Saudi Arabia. The author gave an educational lecture on STIs in a conference room at the prison. Educational material was distributed to the attendees after the lecture, and the survey was conducted one week later. All the participants were asked to complete an anonymous 40-item self-administered questionnaire in the presence of a professional health assistant and a translator, for non-Arabic speakers. Data collected included the personal data of the respondent, her alleged criminal background, penal status, accumulative time in prison, history of smoking, alcohol or drug addiction, knowledge about the seven most common STIs, symptoms, modes of transmission, prevention, sexual activity, addiction, and means of protection. Descriptive analysis was performed using Microsoft Excel.

**Results:**

We interviewed 204 women aged 16-60 years (mean, 33.3 years). Most of the respondents (n = 170; 83 · 0%) were not aware of STIs; 117 respondents (57 · 4%) did not undergo screening for STIs before marriage or intercourse, while only 59 (28 · 9%) did. Over half of the respondents (n = 107; 52.5%) thought they knew how to protect themselves from STIs. Nevertheless, 87 (42.6%) were uncertain about the role of condoms in protection from STIs and (n = 41; 20.1%) thought condoms provide 100% protection against STIs, while 72 respondents (35.3%) thought condoms did not confer 100% protection against STIs. Only 10 respondents (4.9%) used condoms to protect themselves from STIs. Saudi women (P = 0.033) and those with a higher level of education (P < 0.01) were significantly more likely to have better knowledge.

**Conclusion:**

Women inmates at Briman Prison have poor knowledge of STIs as well as risky sexual behaviors. Campaigns aimed at increasing awareness of STIs should also target prison inmates, who in general constitute high-risk populations.

## Background

Over the recent years, there has been an increase in the spread of human immunodeficiency virus (HIV) infection and other sexually transmitted infections (STIs) in many countries [1]. Because of the stigma associated with these infections, multiple barriers to testing and treatment exist, mainly in conservative communities. In order to fight the spread of HIV and STIs, many countries have adopted different modalities to improve awareness. Campaigns have proven effective in achieving better awareness, increasing health-seeking behavior, testing for STIs, and HIV/STI knowledge [2].

To reduce the incidence of HIV and STIs, it is necessary to target high-risk populations such as prison inmates. More so, interventions among prison inmates may provide an opportunity to reach high-risk populations and prevent the wider spread of sexually transmitted HIV [3]. In conservative countries, the lack of education on sexual health has made it increasingly difficult to prevent the spread of STIs [4], both in the general population and in at-risk persons.

Very few studies focusing on knowledge and awareness of STIs have been conducted in both adolescents and adults in Saudi Arabia [5,6]. According to findings from a report that evaluated awareness of sexually transmitted infections (STIs) among Saudi adolescents, most respondents were superficially aware of STIs, and had incorrect perceptions about the mode of transmission and means of protection [4]. This study was designed to explore the range of knowledge on HIV and STIs, sexual behaviors, and adoption of preventive measures among women inmates at Briman Prison, Jeddah.

## Methods

We conducted a survey between July 1, 2012 and July 29, 2012 among women inmates who were detained at Briman Prison, Jeddah. The Institutional Review Board of the Ministry of Health (Western Province, Saudi Arabia) provided ethical review for the survey, including voluntary recruitment, strict confidentiality provisions, and informed consent.

The author gave an education lecture to nearly 300 women prisoners at a conference room in Briman Prison. Educational material was distributed to the attendees at the end of the lecture. The author clearly explained that participation in the survey was completely voluntary, and it was limited to those who were interested. The survey was conducted one week later to allow time for the women to think about participating. Of the 300 women who attended the lecture, only 201 decided to participate, representing a response rate of 67%.

Participants were informed that they were not obliged to answer any question if they were not comfortable answering it. The prison supervisor was not present in the section allocated for our study so as to prevent any feeling of pressure on the participants, who were actually discouraged by the prison administration from participating. Participants were asked to complete a questionnaire in the presence of a professional health assistant and a translator, for non-Arabic speakers. The interviewers explained the purpose of the research to all participants, who were asked to fill a 40-item questionnaire. Special assistance was provided to the illiterate. The questionnaire was developed in Arabic by selecting items based on expert opinion, taking into account the subjective perception of the respondents’ knowledge of STIs. The questionnaire was divided into six sections. (1) Personal data of the respondent, including educational status, marital status, financial situation, and means of support. (2) The alleged criminal background of the respondent, penal status, accumulative time in prison. (3) Sexual activity and smoking, alcohol or drug addiction (4) Knowledge about the seven most common STIs (human immunodeficiency virus, herpes simplex virus type 2, human papilloma virus, syphilis, chlamydia, gonorrhea, and hepatitis B virus), symptoms, modes of transmission, complications, prognosis, treatment, and prevention. (5) Source of information (6) Protection against STIs, analysis of condom usage, and actions taken if partner acquired one of the STIs. The questionnaire questions are presented below:

● 1-Age, 2-Gender, 3-Ethnicity, 4-Religion, 5-Number of children

● 6-Mode of Living (1-apartment rented 2- apartment owned 3-rented villa 4- villa owner)

● 7-Educational attainment (1-uneducated 2-primary education 3-secondary education 4- college graduate)

● 8- Marital status/number of previous marriages/relationships?

● 9-What were you convicted of? What is your penal status?

● 10- Do you work?

● 11-Does your husband work?

● 12- Are you financially dependent on your husband?

● 13-Have you undergone premarital checkup?

● 14-Do you smoke?

● 15-Do you drink alcohol?

● 16-Do you use drugs? If YES, specify:

● Rush, Hashish, Captagon, Heroin, Logga, Powder, Cocaine, Amphetamine, Chewing tobacco (noshog), Morphine, Sniffing glue (Ghera’a)

● Do you know the mode of transmission of the following infections?

● 17-HSV, 18-HPV, 19-SYPHILLIS, 20-GONORRHEA, 21- CHLAMYDIA, 22-HIV, 23-HEPATITS-B, 24-HIV

● 25-Is there a vaccine to protect you against ALL STIs?

● Do you know the clinical presentation of these infections?

● 26- Herpes simplex virus? (1-warts 2-ulcers 3-vaginal discharge 4-papules 5-I don’t know)

● 27- Human papilloma virus? (1-warts 2-ulcers 3-vaginal discharge 4-papules 5-I don’t know)

● 28-What is your source of information?

● TV/Radio, Internet, Books, Magazines/Newspaper, Friends, Family, Others

● 29-Do you know how to protect yourself from STIs?

● 30-Do you think condoms are 100% protective against STIs?

● 31-Would you like to know if you already acquired any STIs?

● 32-Do you think you have the right to know if your husband has any STIs?

● 33-What will you do if you found out your husband has acquired one of the STIs?

● 34- ask for divorce, 35- ask him to seek medical advice, 36- seek medical advice, 37- will not do anything, 38- will refrain from sexual intercourse, 39- not sure

● 40-Do you think you should consider studying these infectious diseases in school or during secondary education?

### Statistical analysis

A simple descriptive statistics was performed to show the frequency distributions, mean, range and variations using Microsoft Excel 2007®. Chi-square test was used to establish a relationship between two categorical variables. To measure the knowledge, a mean score was calculated from a number of criterion and measures were grouped into two categories. Lastly, a conventional p-value <0.05 was used to reject null hypothesis.

## Results

We interviewed 204 women prisoners aged 16–60 years (mean, 33.3 years ± 9.3 years). Most of the respondents were convicted for prostitution (n = 105; 51.4%), followed by illegal immigration 20(9.8%) and wine trading (n = 14; 6.8%); adultery, begging, and murder were less frequent reasons for incarceration (Table 1). Of the 204 respondents, there were 65 Indonesians (31.9%), 35 Somalis (17.2%), 32 Ethiopians (15.7%), and 16 Saudis (7.8%); the remaining 56 respondents were from neighboring countries. Regarding marital status, 80 women (39.2%) were married, 62 (30.4%) were single, 43 (21.0%) were divorced and 19 (9.3%) were widows. 70 (34.3%) of the respondents were illiterate; 67 (32.9%) had completed secondary education, while 56 (27 · 5%) had only completed primary education. Only 10 (4.9%) were college graduates. One respondent did not reveal her level of education. Among the respondents, 146 (71.5%) were working prior to imprisonment, whereas 81 (39.7%) were financially dependent on their partners.

**Table 1:** Offences Committed by the respondents

**Type of crime**	**Number of prisoners**
Prostitution	105 (51.4%)
Wine trading	14 (6.8%)
Forgery, blackmail, theft	10 (4.9%)
Witchcraft	2 (0.9%)
Fighting	7 (3.4%)
Drug dealing	8 (3.9%)
Illegal immigration	20 (9.8%)
Financial matters/debt	4 (1.9%)
Adultery	9 (4.41%)
Murder	5 (2.4%)
Begging	5 (2.4%)
Others	15 (7.3%)

Thirty-eight respondents (18.6%) were smokers, with no history of alcohol addiction. A history of drug abuse was recorded in 4 respondents (1.9%).

While 117 respondents (57.4%) reported that they did not undergo screening for STIs before marriage or sexual intercourse, 59 (28.9%) reported they did; 28 respondents (13.7%) declined to respond. Over half of the respondents (n = 107; 52.5%) thought they knew how to protect themselves from STIs, while 92 (45.1%) reported having no knowledge on protective methods. 3 respondents (1.5%) were uncertain, while 5 (2.4%) declined to respond.

One in five of the respondents (n = 41; 20 · 1%) thought condoms provide 100% protection against STIs. Eighty-seven (42.6%) were uncertain about the role of condoms in protection from STIs while 72 respondents (35.3%) thought condoms did not confer 100% protection against STIs. 4 (2.0%) respondents declined to give their opinion on the use of condoms. Only 10 respondents (4.9%) reported they used condoms to protect themselves from STIs, while 31 (15.2%) reported they have used it for contraceptive purposes. Only 3 out of the 31 women who used condoms for contraceptive purposes have used it for protection from STIs as well. Nineteen women (9.3%) declined to respond.

Most of the respondents (n = 170; 83 · 0%) were not aware of STIs. Friends, television, and radio programs were the major sources of information (n = 60; 29 · 4%, in each case). Other sources of information included family members (n = 46; 22 · 5%), newspapers and magazines (n = 22; 10 · 8%), books (n = 20; 9 · 8%), and the Internet (n = 20; 9 · 8%). Only 20 respondents (9 · 8%) mentioned other less common sources of information.

Women with a higher the level of education were significantly more likely to have better knowledge about STIs (P-value <0.001; Table 2). Saudi women also seemed to have better knowledge than expatriate women (P = 0.033). Table 2 demonstrates the effect of different variables on the level of awareness. Their level of knowledge was considered to be good if they scored ≥ 50% and poor if they scored < 50% (Tables 3 and 4).

**Table 2:** Correlation between different variables and level of awareness about sexually transmitted infections

**Variables**			**Knowledge group**		**Total**	***X***^**2**^	**P-value**
			**<50%**	**≥50%**			
			**Correct**	**Correct**			
**Age**	≤30	Count	91	7	98	1.510	0.219
		%	92.9%	7.1%	100.0%		
	>30	Count	93	13	106		
		%	87.7%	12.3%	100.0%		
**Total**		Count	184	20	204		
		%	90.2%	9.8%	100.0%		
**Nationality**	Saudi	Count	12	4	16	4.534	0.033*
		%	75.0%	25.0%	100.0%		
	Non-Saudi	Count	172	16	188		
		%	91.5%	8.5%	100.0%		
**Total**		Count	184	20	204		
		%	90.2%	9.8%	100.0%		
**Religion**	Islam	Count	174	18	192	1.584	0.208
		%	90.6%	9.4%	100.0%		
	Christian	Count	7	2	9		
		%	77.8%	22.2%	100.0%		
**Total**		Count	181	20	201		
		%	90.0%	10.0%	100.0%		
**Marital status**	Single	Count	54	8	62	7.199	0.066
		%	87.1%	12.9%	100.0%		
	Married	Count	69	11	80		
		%	86.3%	13.8%	100.0%		
	Divorced	Count	43	0	43		
		%	100.0%	0.0%	100.0%		
	Widowed	Count	18	1	19		
		%	94.7%	5.3%	100.0%		
**Total**		Count	184	20	204		
		%	90.2%	9.8%	100.0%		
**Housing**	Rental	Count	162	16	178	1.398	0.237
		%	91.0%	9.0%	100.0%		
	Owned	Count	20	4	24		
		%	83.3%	16.7%	100.0%		
**Total**		Count	182	20	202		
		%	90.1%	9.9%	100.0%		
**Educational attainment**	Primary or below	Count	122	4	126	36.582	<0.001*
		%	96.8%	3.2%	100.0%		
	Secondary	Count	57	10	67		
		%	85.1%	14.9%	100.0%		
	University	Count	4	6	10		
		%	40.0%	60.0%	100.0%		
**Total**		Count	183	20	203		
		%	90.1%	9.9%	100.0%		
**Employment status**	Yes	Count	132	14	146	0.027	0.870
		%	90.4%	9.6%	100.0%		
	No	Count	52	6	58		
		%	89.7%	10.3%	100.0%		
**Total**		Count	184	20	204		
		%	90.2%	9.8%	100.0%		

*Significant using chi-square test > 0.05 level.

**Table 3:** Scoring system regarding level of knowledge

**Variables**		**Frequency**	**Percent**
Knowledge group	<50% correct	184	90.2
	≥50% correct	20	9.8
	Total	204	100.0

**Table 4:** Level of knowledge on the seven sexually transmitted infections

**Knowledge variables**		**Frequency**	**Percent**
Symptoms of herpes simplex virus	Correct	13	6.4
	Incorrect	191	93.6
	Total	204	100.0
Symptoms of human papilloma virus	Correct	4	2.0
	Incorrect	200	98.0
	Total	204	100.0
Do you think condoms provide 100% protection against sexually transmitted infections?	Correct	72	35.3
	Incorrect	132	64.7
	Total	204	100.0
Do you think the following infections are transmitted by sexual contact?			
Herpes simplex virus	Correct	24	11.8
	Incorrect	180	88.2
	Total	204	100.0
Human papilloma virus	Correct	18	8.8
	Incorrect	186	91.2
	Total	204	100.0
Syphilis	Correct	26	12.7
	Incorrect	178	87.3
	Total	204	100.0
Gonorrhea	Correct	19	9.3
	Incorrect	185	90.7
	Total	204	100.0
Chlamydia	Correct	15	7.4
	Incorrect	189	92.6
	Total	204	100.0
Human immunodeficiency virus	Correct	91	44.6
	Incorrect	113	55.4
	Total	204	100.0
Hepatitis B virus	Correct	21	10.3
	Incorrect	183	89.7
	Total	204	100.0

Over half of the respondents (n = 159; 77 · 9%) reported they would want to know whether they had any of the STIs. 172 respondents (84 · 3%) thought they had the right to know if their spouses had an STI; 180 (88 · 2%) believed their spouses had the right to know if they had an STI. When asked about their reaction to a spouse testing positive for any of the STIs, 181 respondents (88 · 7%) responded they would ask their spouses to seek medical advice, 67 (32 · 8%) reported they would file for divorce, and only 66 (32 · 4%) responded they would seek medical check-up.

Regarding the respondents’ opinion about awareness and prevention of STIs, 31 (15 · 2%) responded that this information should be taught in primary or secondary school.

## Discussion

This study is novel in that it is the first to explore the sexual behaviors and degree of knowledge about STIs among women prisoners in Saudi Arabia. Our findings revealed that women inmates had poor knowledge of STIs, which could explain their risky sexual behaviors. Over half of the respondents had never undergone screening for STIs before marriage or sexual intercourse, and up to 92 respondents (45.1%) had no knowledge on how to protect themselves from STIs. In addition, 41 (20 · 1%) of the respondents in our study believed that condoms gave 100% protection against STIs, and 87 (42 · 6%) were unsure of their protective effect.

Contrary to reports from other authors who reported that 44 · 0% of the respondents in their study used condoms consistently [7], we found that only 10 (4 · 9%) of the respondents in our study used condoms for protection against STIs and 31 (15 · 2%) used them for contraceptive purposes. However, this observation could be due to the fact that up to 39 · 2% of the respondents in our study group were married and 51 · 5% were allegedly incarcerated for prostitution. There are reports that sex workers generally have high-risk sexual behaviors, and the consumption of alcohol prior to sex among migrant female sex workers has been reported to be significantly associated with inconsistent condom use during paid or unpaid sex [8]. We did not, however, have any report of alcohol consumption among the respondents in our study, and other factors, such as poor knowledge may explain the low use of condoms among the women.

Clinical studies on the effectiveness of condoms against most STIs suggest inconsistent levels of protection. Studies show varied rates of reduction of HIV transmission in association with condom use. Some advocate the reduction rate of HIV transmission related to condom use by 87% [9]; however, others found that the reduction rate is only 30% in HIV-2 [10]. This is mainly because a condom’s primary role is to create a barrier between sexual fluids. This largely decreases the chances of transmission of STIs that are fluid mediated but can do little against the ones that are transmitted through skin to skin contact or skin to mucosa contact such as HIV-2, human papilloma virus and herpes simplex-2. This is not to say that the use of condoms as a means of protection should be overlooked; however, it is not a foolproof solution. The best prevention method is to refrain from risky sexual activities.

Despite the fact that uneducated women accounted for only 34 · 3% of our study sample, and 32 · 9% had completed at least secondary education, most of the women had risky sexual behaviors. Studies on adults show that there are inconsistent findings on the association between education and sexual risk [11]. In another study involving a sample of incarcerated women aged ≥ 18 years, it was reported that women with more years of education were less likely to engage in risky sexual behaviors, such as having unprotected sex compared with their less educated peers [12]. This contradicts the findings of a meta-analysis that encompassed 28 countries both within and outside of Africa, which demonstrated a rather high prevalence of higher-risk sexual behavior among the wealthier and more educated women. This finding was inspite of the geographic location of the selected low to middle income countries [13].

In Saudi Arabia, there is limited data on STIs. In addition to the fact that many people with STIs are asymptomatic and cases remain undiagnosed, those that are diagnosed are frequently not reported. Underreporting of STIs is not only a problem in Saudi Arabia; it is common in other developed countries as well [14,15]. It is also frequent among female sex workers, mainly due to unawareness of the symptoms of STIs (including asymptomatic STI) and their belief that they are at low-risk for STIs [15]. In our study, 20% of the respondents did not want to know whether they had an STI, which is a relatively high percentage for a high-risk group. Nearly nine out of ten respondents reported they believed that they had the right to know if their partners had an STI. A similar percentage believed their partners had the right to know if they were infected. This might reflect either honesty or lack of understanding on the part of the respondents about the mode of transmission of STIs. When asked about their reaction to a spouse testing positive for any STI, nine out of ten respondents responded that they would ask the partner to seek medical advice, while only one third responded that they would have medical checkup. This as well also reflected deficient knowledge on specific STIs and their respective symptoms, the mode of transmission, and consequences. This lack of awareness and understanding puts their health at risk.

The subject of sexual intercourse is generally a taboo in Saudi Arabia. Until late 2012, providing formal sexual education was not introduced in the curriculum of most private and public schools. Most Saudi adolescents discuss sexual-related issues with their friends, and very few discuss them with a parent or maid [4,5]. Reports from a local study conducted on 417 adolescent girls in public and private schools revealed that less than half of the respondents knew that syphilis, gonorrhea, and hepatitis were STIs [5]. In the current study, up to 170 (83 · 0%) of the respondents were not aware of STIs. The major sources of information included friends, television, and radio programs. Given that most of the respondents in our study were mostly expatriates, the poor knowledge on sexual health observed in this study may indicate that Saudi Arabia and countries such as Indonesia, Somalia and Ethiopia are deficient in providing formal education on sexual health.

Appropriate preventive strategies against STIs that conform to Islamic rules and values are necessary, and these should be of the highest priority for policy makers because of the high probability of spread of infection, particularly amongst young adults [14]. Given the annual influx of large numbers of expatriates into Saudi Arabia and the fact that they could be actively involved in the spread of STIs, it is necessary for Saudi authorities to organize campaigns aimed at educating the general population about common STIs and preventive measures.

This study had some limitations in that the study relied on a self-report method of data collection. More so, bias may have been introduced due to intentional deception, poor memory, or misunderstanding of a question or set of questions. It should be taken into account that a relatively large proportion of the study population—one third (34.3%)—was illiterate, and because the questionnaire was distributed after a lecture, this may have affected the participants’ knowledge. Although Saudi women seemed to have better knowledge than expatriate women, we could not deduce relevant conclusions from this fact owing to the limited number of Saudis in our sample. Nevertheless, this study is the first of its kind to examine sexual behaviors and STI knowledge among women inmates at a prison in Jeddah, Saudi Arabia.

## Conclusion

Women inmates at Briman Prison have poor knowledge of STIs as well as risky sexual behaviors. Lack of STI knowledge puts them particularly at risk of acquiring these infections. Campaigns aimed at increasing awareness of STIs should also target prison inmates, who in general constitute high-risk populations.

## Competing interests

The author declares that they have no competing interests.

## Pre-publication history

The pre-publication history for this paper can be accessed here:

http://www.biomedcentral.com/1471-2334/14/290/prepub
